# Comparison of Ionomic and Metabolites Response under Alkali Stress in Old and Young Leaves of Cotton (*Gossypium hirsutum* L.) Seedlings

**DOI:** 10.3389/fpls.2016.01785

**Published:** 2016-11-25

**Authors:** Rui Guo, LianXuan Shi, ChunWu Yang, ChangRong Yan, XiuLi Zhong, Qi Liu, Xu Xia, HaoRu Li

**Affiliations:** ^1^Key Laboratory of Dryland Agriculture, Institute of Environment and Sustainable Development in Agriculture, Chinese Academy of Agricultural SciencesBeijing, China; ^2^Key Laboratory of Molecular Epigenetics of Ministry of Education, Northeast Normal UniversityChangchun, China

**Keywords:** cotton, alkali stress, young leaves, old leaves, growth, metal elements, free ions, metabolites

## Abstract

Soil salinization is an important agriculture-related environmental problem. Alkali stress and salt stress strongly influence the metabolic balance in plants. Salt and alkali stresses exert varied effects on old and young tissues, which display different adaptive strategies. In this study, we used cotton (*Gossypium hirsutum* L.) plants as experimental material to investigate whether alkali stress induces ionic and metabolism changes in old and young leaves of cotton plants exposed to alkali stress. Results showed that alkali stress exerted a considerably stronger growth inhibition on old leaves than on young leaves. Under alkali stress, young leaves can maintain low Na and high K contents and retain relatively stable tricarboxylic acid cycle, resulting in greater accumulation of photosynthetic metabolites. In terms of metabolic response, the young and old leaves clearly displayed different mechanisms of osmotic regulation. The amounts of inositol and mannose significantly increased in both old and young leaves of cotton exposed to alkali stress, and the extent of increase was higher in young leaves than in old leaves. In old leaves, synthesis of amino acids, such as GABA, valine, and serine, was dramatically enhanced, and this phenomenon is favorable for osmotic adjustment and membrane stability. Organs at different developmental stages possibly display different mechanisms of metabolic regulation under stress condition. Thus, we propose that future investigations on alkali stress should use more organs obtained at different developmental stages.

## Introduction

Soil salinization is an important agriculture-related problem, which frequently co-exists with salt and alkali stresses (Wang et al., 2011). Approximately 831 mha soils worldwide is saline, 434 and 397 mha of which have been affected by alkali and salt stresses, respectively (Wang et al., 2012). More than 70% of the land area in northeast China is alkali grassland characterized by high pH, and only a few alkali-tolerant halophytes can survive in these areas (Kawanabe and Zhu, 1991; Zheng and Li, 1999). Previous studies have shown that salt and alkali conditions in the rhizosphere induce two distinct forms of stress in plants. For this reason, the effect of these stresses on plants should be investigated using different approaches, salt stress was simulated by mixing neutral salts (NaCl and Na_2_SO_4_) and alkali stress was simulated by mixing alkaline salts (NaHCO_3_ and Na_2_CO_3_; Shi and Wang, 2005). Some reports have shown that alkali stress causes considerably stronger destructive effects on plants than salt stress; however, the amount of attention devoted to alkali stress remains low (Yang et al., 2008).

Numerous studies have reported on the effect of salt stress on old and young tissues, and their results showed that salt stress exerts distinct effects on growth, ion balance, compatible solutes, and metabolism in old and young leaves (Nakamura et al., 1996; Ashraf and O’Leary, 1997; Hajlaoui et al., 2010). Salt stress is caused by neutral salts and generally induces osmotic stress and ion-induced injury (Shi and Wang, 2005). Compared with salt stress, alkali stress is caused by alkaline salts; alkali stress demonstrates stress factors similar to those of salt stress but it becomes aggravated when combined with high pH stress (Ghoulam et al., 2002; De-Lacerda et al., 2003). A high-pH environment around the roots reduces the availability of mineral elements for example, by precipitating Ca^2+^, Mg^2+^, and HPO_3_^-^, as well as inhibiting the absorption of inorganic anions, thereby disrupting ionic balance in tissues (Yang et al., 2008, 2009). Moreover, alkali stress can destroy root membrane structure and strongly affect the normal physiological functions of roots (Yang et al., 2008, 2009). Several studies have focused on alkali stress; however, a comparative study on the effect of alkali stress on old and young tissues is lacking (Gao et al., 2008; Wang et al., 2008; Yang et al., 2008). During plant adaptation to abiotic stress, old and young leaves may display distinct regulatory mechanisms and play different roles in alkali tolerance. Therefore, understanding the distinct effects of alkali stress on old and young leaves is potentially important in elucidating the adaptation and tolerance of plants to alkali stress.

Maintenance of ion homeostasis and pH are crucial for alkali tolerance in plants (Yang et al., 2008, 2009). Na^+^ and K^+^ transport, Na^+^ exclusion, and pH adjustment are key adaptive factors needed by a plant to adapt to alkali stress (Shi et al., 2002; Munns and Tester, 2008). Some Na^+^ and K^+^ transporters have been identified, and salt overly sensitive pathway plays an important role in Na^+^ reduction (Shi et al., 2002; Munns and Tester, 2008). Plants can secrete high concentrations of metabolites to adjust the pH of tissues, and this process is the central mechanism by which plants resist alkali stress (Wang et al., 2012a,b). However, the responses of other ions, metabolites, and their interactions to alkali stress have not yet been fully elucidated. Ionomics is the inorganic component of cellular and organismal systems, including mineral nutrient and trace element compositions of tissues (Wu et al., 2013). In rice (*Oryza sativa L.*), alkali stress strongly affects the uptake and accumulation of some metabolites, such as nitrogen and proline; these changes in composition in response to physiological processes are possibly important in alkali tolerance in plants (Yang et al., 2008, 2009). Wang et al. (2012b) showed that during adaptation of rice to alkali stress, young and old leaves have distinct mechanisms of ion balance and nitrogen metabolism regulation. However, the relationship between ionic response and metabolite accumulation in old and young leaves under alkali stress has not yet been investigated.

Cotton (*Gossypium hirsutum* L.) is an important industrial crop exhibiting wide adaptability, and some cultivars are tolerant to salt and water stresses; for this reason, cotton is frequently used as model crop to understand salinity tolerance. In addition, cotton is adapted to a broad range of environmental conditions and has formed rich genetic diversities for salt tolerance (Zhang X. et al., 2011; Wang et al., 2012). In this study, we used cotton plants as experimental material to investigate whether alkali stress exerts different effects on ion balance and metabolism in old and young leaves of cotton.

## Materials and Methods

### Plant Materials and Growth Conditions

The seeds of cotton (*G. hirsutum* L.) Yiluzao-7, a major cotton cultivated in north China, were sown in 34 cm diameter plastic pots containing 5.5 kg of washed sand. Each pot contained five seedlings. The pots were watered daily with Hoagland nutrient solution at 17:00–18:00. All pots were placed outdoors but were kept from rain. The temperature range at day time was 23–27°C and 19–22°C at night time.

### Stress Treatments

Two alkali salts (NaHCO_3_ and Na_2_CO_3_) were selected based on the salt content and pH of the majority of alkali soils in northeast China. For the alkali stress treatment, two alkali salts were mixed in a 9:1 molar ratio (NaHCO_3_:Na_2_CO_3_). The total salt concentration was set at 80 mM (pH, 9.06). In the 80 mM alkali stress treatment solution, a mixture of 72 mM NaHCO_3_ and 8 mM Na_2_CO_3_ resulted in ion concentrations of 88 mM Na^+^, 72 mM HCO_3_^-^, and 8 mM CO_3_^2-^.

Firstly, all plants were grown in full Hoagland nutrient solution for 30 days (since sowing). After growing for 30 days, ten pots of seedlings were randomly divided into two sets: one set (five pots) was used as control, which was watered with nutrient solution daily for three times; another set (five pots) was watered with the nutrition solution with the alkali salts daily for three times. The plants were subjected to alkali stress solution by watering from 17:00 to 18:00 for six consecutive days. Five seedlings of a pot were pooled as a biological replicate, therefore, each treatment have five pots as five biological replicates. The five seedlings in each pot were harvested after treatment for 6 days.

### Measurement of Photosynthetic Pigments

Chlorophylls *a* and *b* and carotenoid were extracted with acetone; each sample was spectrophotometrically analyzed for five times at 440, 645, and 663 nm five times in accordance with previously described methods. We used the equations proposed by Arnon (1949) for pigment concentration estimations.

### Measurement of Metal Elements and Inorganic Anions

Dry old and young leaves were ground, and ∼100 mg of tissue samples were dry-digested in a muﬄe furnace at 500°C for 6 h, and then 10 mL of HNO_3_:H_2_O (1:1) was added to extract ions. The contents of Na, K, Ca, Mg, P, Fe, Cu, Zn, and Mn were determined using an ICP-OES spectrometer (iCAP 6000 series, Thermo Fisher Scientific Inc.) according to the operation manual. After water extraction was performed, the quantities of anions (NO_3_^-^, Cl^-^, SO_4_^2-^, and H_2_PO_4_^-^) were determined through ion exchange chromatography (DX300 ion chromatographic system; AS4A-SC ion-exchange column, CD M-II electrical conductivity detector; DIONEX, Sunnyvale, CA, USA) with a mobile phase comprising 1.7 mM/1.8 mM Na_2_CO_3_/NaHCO_3_. Each measurement was repeated five times.

### Measurement of Metabolites

Approximately 100 mg of each frozen tissue sample was transferred into 2 ml centrifuge tubes, and 60 μl of water containing ribitol as an internal standard was added to each tube. After the mixtures were vortexed with 0.3 ml of methanol and 0.1 ml of chloroform, a 70 Hz grinding mill system (Jinxin Biotech LTD. Shanghai, China) was used to grind the samples for 5 min; the samples were then incubated at 70°C for 10 min. The tubes were centrifuged at 12,000 rpm at 4°C for 10 min. Supernatant (0.35 ml) was decanted into a 2 ml volume screw-top glass tube; the samples were dried in a vacuum concentrator at 30°C for 2 h. Afterward, each sample was dissolved in 80 μl of methoxamine hydrochloride and incubated at 37°C for 2 h. The samples were further derivatized with N,O-bis(trimethylsilyl)-trifluoroacetamide (BSTFA) containing 1% trimethylchlorosilane (TMCS; 100 μl) at 70°C for 1 h (Lisec et al., 2006). GC-TOF/MS analysis was performed using a 1D Agilent 7890 gas chromatograph system coupled with a Pegasus 4D time-of-flight mass spectrometer. The system was equipped with a DB-5MS capillary column coated in 5% diphenyl cross-linked with 95% dimethylpolysiloxane (30 m × 250 μm inner diameter and 0.25 μm film thickness; J&W Scientific, Folsom, CA, USA). An aliquot of the analyte (1 μL) was injected in a splitless mode. Helium was used as carrier gas, the front inlet purge flow was 3 mL min^-1^, and the gas flow rate through the column was 1 mL min^-1^. The initial temperature was maintained at 90°C for 0.25 min; temperature was increased to 180°C at a rate of 10°C min^-1^ and to 240°C at a rate of 5°C min^-1^. Temperature was further increased to 285°C at a rate of 20°C min^-1^ for 11.5 min. Injection, transfer line, and ion source temperatures were 280, 270, and 220°C, respectively. Energy was set at -70 eV in an electron impact mode. Mass spectrometry data were acquired in a full-scan mode with an m/z range of 20–600 at a rate of 100 spectra per second after a solvent delay of 492 s.

### Statistical Analysis

Photosynthetic pigments, metal elements, and inorganic anions were statistically analyzed using SPSS 13. All data were presented as average, along with the standard error (SE), of five biological replicates. Significant difference between old and young leaves at same stress condition was determined by *T*-test. Metabolites were identified by searching FiehnLib (GC-TOF), a commercial EI-MS library (Kind et al., 2009). The resulting three-dimensional data, namely, peak number, sample name, and normalized peak area, were run in SIMCA-P 13 software package (Umetrics, Umea, Sweden) and subjected to principal component analysis (PCA) and orthogonal projections to latent structure-discriminant analysis (OPLS-DA). Non-commercial databases, including Kyoto Encyclopedia of Genes and Genomes (KEGG)^1^, were utilized to search for metabolite pathways. Format data were uploaded in the MetaboAnalyst website ^2^ for further analysis (Paz and Martinez-Ramos, 2003).

## Results

### Growth and Ion Accumulation Responses of the Seedlings to Alkali Stress

**FIGURE 1:**
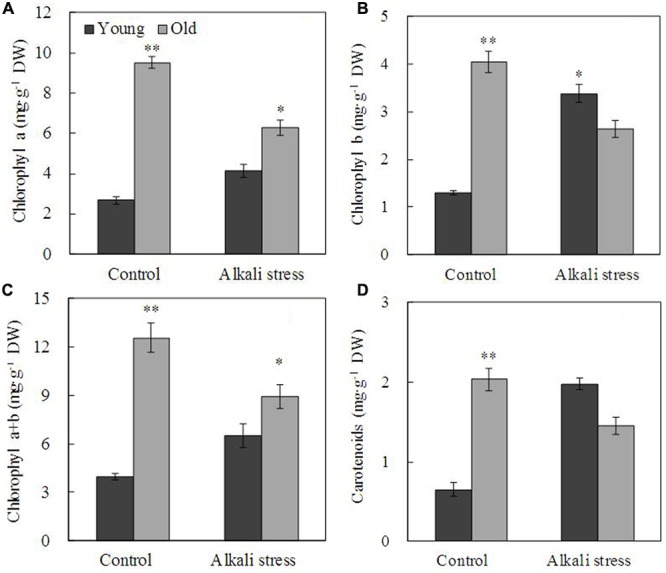
**Effects of alkali stress on the contents of pigment chlorophyll a (A)**, chlorophyll b **(B)**, chlorophyll a+b **(C)** and carotenoid **(D)** in young and old leaves of cotton seedlings. The values are the means ± SE of five biological replicates, and each replicate consisted of a pool of five plants. Significant difference between old and young leaves under similar stress condition was determined by t-test and marked as ^∗^(P < 0.05) and ^∗∗^(P < 0.01). The seedlings were subjected to 80 mM alkali stress (NaHCO_3_:Na_2_CO_3_ = 9:1; pH = 9.06) for 6 days.

**FIGURE 2:**
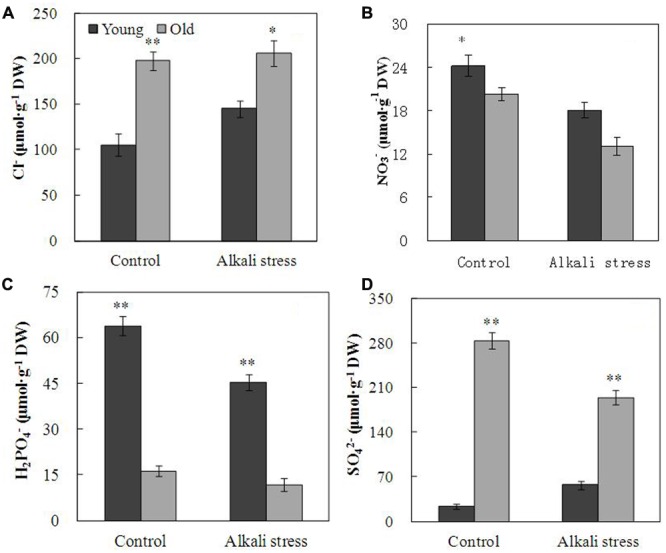
**Effects of alkali stress on the contents of inorganic ion Cl- (A)**, NO_3_^-^ (B), H_2_PO_4_^-^ **(C)**, and SO_4_^2-^ **(D)** in young and old leaves of cotton seedlings. The values are the means ± SE of five biological replicates, and each replicate consisted of a pool of five plants. Significant difference between young and old leaves under similar stress condition was determined by t-test and marked as ^∗^(P < 0.05) and ^∗∗^(P < 0.01). The seedlings were subjected to 80 mM alkali stress (NaHCO_3_:Na_2_CO_3_ = 9:1; pH = 9.06) for 6 days.

Alkali stress-induced growth inhibition was more evident in old leaves than in young leaves (**Figure 1**). In addition, alkali stress increased the concentrations of chlorophylls *a, b, a*+*b*, and carotenoids in young leaves but it reduced their amounts in old leaves (**Figure 1**). In response to 6-day alkali stress treatment, Na content in old and young leaves significantly increased by 8.67 and 3.39 times, respectively (**Table 1**). K content in old leaves decreased, whereas no significant change was observed in young leaves (**Table 1**). The effects of alkali stress on Ca, Mg, Fe, Cu, Zn, and Mn were not significant both in old and young leaves (**Table 1**). Moreover, under alkali stress, Ca and K became the dominant component in the old and young leaves of total mineral element, respectively (**Table 1**). Alkali stress increased Cl^-^ contents in both old and young leaves but it reduced NO_3_^-^ and H_2_PO_4_^-^ contents (**Figure 2**). Moreover, SO_4_^2-^ contents increased in young leaves but decreased in old leaves under alkali stress (**Figure 2**).

**Table 1:** Effects of alkali stress on the amounts of mineral elements in young and old leaves of cotton seedlings.

Treatment		Control		Alkali stress	
Samples		Young	Old	Young	Old
Mineral Element	Na	20.04 ± 0.38	21.90 ± 4.22^∗^	87.89 ± 2.63^∗∗^	211.74 ± 13.30^∗∗^
	K	513.29 ± 23.84	470.60 ± 19.72^∗^	477.26 ± 21.05	386.94 ± 28.34
	Ca	217.61 ± 16.65	857.51 ± 29.43^∗∗^	183.24 ± 14.24	864.38 ± 33.28^∗∗^
	Mg	74.89 ± 6.78	186.21 ± 10.72^∗∗^	70.24 ± 8.33	187.13 ± 11.92^∗∗^
	Fe	1.94 ± 0.18	3.81 ± 0.27^∗^	2.11 ± 0.18	3.33 ± 0.31^∗^
	Cu	0.22 ± 0.02	0.21 ± 0.02	0.60 ± 0.03^∗^	0.31 ± 0.03
	Zn	0.55 ± 0.03^∗^	0.26 ± 0.03	0.66 ± 0.02^∗∗^	0.22 ± 0.02
	Mn	0.48 ± 0.02	1.40 ± 0.12^∗∗^	0.51 ± 0.02	1.55 ± 0.13^∗∗^

*The values are the means ± SE of five biological replicates, and each replicate consisted of a pool of five plants. Significant difference between organs under similar stress condition was determined by *T*-test and marked as ^∗^*P* < 0.05 and ^∗∗^*P* < 0.01. Significant difference between control and alkali stress in the same organs was determined by *T*-test and marked as ^∗^*P* < 0.05 and ^∗∗^*P* < 0.01. The seedlings were subjected to 80 mM alkali stress (NaHCO_3_:Na_2_CO_3_ = 9:1; pH = 9.06) for 6 days.*

### Metabolite Responses to Alkali Stress at the Seedling Stage

**FIGURE 3:**
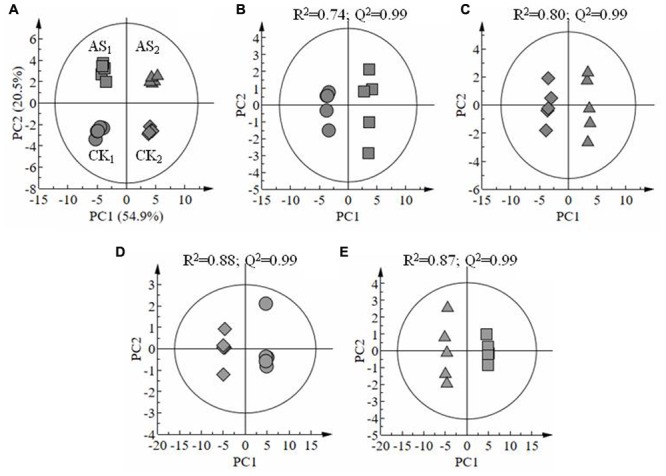
**Score plots of principal component analysis results showing the metabonomic trajectory of old and young leaves of cotton seedlings treated with and without alkali stress **(A)**.** Orthogonal partial least-squares discriminant analysis scores showing the dose-dependent effect of stress on cotton leaves: CK_1_ vs. AS_1_ **(B)**, CK_2_ vs. AS_2_ **(C)**; CK_1_ vs. CK_2_ **(D)** and AS_1_vs. AS_2_ **(E)**. Old (CK_1_) and young (CK_2_) leaves of cotton treated without stress; Old (AS_1_) and young (AS_2_) leaves under alkali stress treatment.

A total of 133 metabolites were identified in the leaves of cotton seedlings, and all samples were distributed within the 95% confidence interval of Hotelling’s T2 ellipse (**Figure 3**). The scores plot of PCA results showed that ∼75% variability in the four groups of samples can be explained using two principal components (**Figure 3A**). Additionally, heatmap analysis (**Supplementary File S2**) showed an obvious separation between samples treated with and without alkali stress treatment in different tissues. OPLS-DA was conducted using one orthogonal and one predictive component calculated from all models derived from two classes of samples. The scores plot of OPLS-DA results clearly showed the separation between the leaves of cotton leaves treated with 80 mM alkali stress for 6 days and with good model quality (**Figures 3B–E**). A total of 133 metabolites, including organic acids, amino acids, sugars and poly-sugars, and nucleotide derivatives, were identified. Detailed information on these identified metabolites is shown in **Supplementary File S1**. Most of the metabolites detected in this study were predominantly classified under the general biochemical pathways, such as TCA cycle, glycolysis, GABA pathway, GS/GOGAT cycle, Shikimic acid pathway, and amino acid metabolism (**Table 2**) based on search results in Plant Metabolic Network^3^ and KEGG.

**Table 2:** Relative concentration and fold changes in major metabolites in young and old leaves of cotton seedlings after 6 days of alkali stress treatment.

Metabolic pathway/compound		Relative concentration				Fold changes	
		Control		Alkali stress		log_2_^AS/CK^	
		Young	Old	Young	Old	Young	Old
*TCA cycle*	Oxalic acid	0.06	0.03	0.05	0.01	-0.32	-0.96ˆ*
	Citric acid	1.21	2.09	1.16	1.02	-0.06	-1.04ˆ*
	Aconitic acid	0.21	0.03	0.10	0.01	-1.02ˆ*	-1.34ˆ**
	α-Ketoglutaric acid	0.28	0.19	0.16	0.10	-0.82	-0.91ˆ*
	Succinic acid	4.67	2.95	2.98	0.94	-0.65	-1.65ˆ**
	Fumaric acid	0.19	0.55	0.19	0.43	0.02	-0.35
	Malic acid	5.15	12.18	6.42	8.27	0.32	-0.56
*Glycolysis*	Pyruvic acid	0.17	0.29	0.49	0.31	0.14	0.09
	PEP	0.01	0.03	0.03	0.02	1.12ˆ*	-0.24
	3PGA	0.16	1.03	0.22	0.65	2.66ˆ**	1.55ˆ*
	Fructose-6-phosphate	0.02	0.05	0.03	0.03	0.98ˆ*	0.24
	Glucose-6-phosphate	0.03	0.07	0.05	0.04	0.95ˆ*	-0.28
	Glucose	0.02	0.22	0.11	0.20	2.18ˆ**	-0.12
	Fructose	1.86	2.40	2.42	2.03	0.38	-0.24
	Sucrose	0.18	0.51	0.14	0.30	-0.36	-0.78ˆ*
*Proline synthesis pathway*	Proline	1.04	2.73	33.99	16.79	5.02ˆ**	2.62ˆ**
	Glutamic acid	0.06	0.08	0.03	0.09	-1.02ˆ*	-0.17
*GS/GOGAT cycle*	Aspartic acid	14.35	16.16	9.94	18.41	-0.53ˆ*	0.19
	Asparagine	0.10	0.12	0.67	0.12	2.68ˆ**	-0.02
	Glutamine	0.19	0.22	0.10	0.15	-1.00ˆ*	-0.58
*GABA path way*	GABA	2.50	6.04	3.96	8.72	0.67ˆ*	0.53ˆ*
	SSA	0.01	0.01	0.01	0.01	0.22	0.52
	Putrescine	0.29	0.45	0.03	0.04	0.64ˆ*	0.28
*Metabolism of plasma membrane*	Glycine	0.18	0.15	0.16	0.29	-0.20	0.89
	Serine	0.67	0.69	0.45	0.92	0.04	1.05ˆ*
	Ethanolamine	4.52	2.03	4.33	0.82	-0.06	-1.32ˆ**
*Shikimic path way*	Shikimic acid	0.95	0.30	0.72	0.15	-0.41	-1.02ˆ*
	Quinic acid	0.57	0.01	1.11	0.02	0.97	1.18ˆ*
	Chlorogenic acid	0.70	1.11	1.27	1.45	0.85	0.39
	Phenylalanine	0.46	0.14	0.65	0.14	0.50	0.04
	Tyrosine	0.00	0.01	0.00	0.01	0.30	-0.03
	Tryptophan	0.43	0.01	0.72	0.02	0.75	0.62
	Cinnamic acid	0.16	0.06	0.24	0.07	0.57	0.14
	Ferulic acid	0.39	0.28	0.45	0.48	0.20	0.79

*Fold changes were calculated using the formula log_2_^(Alkali/control)^. ^∗^indicates significance (*P* < 0.05); ^∗∗^indicates high significance (*P* < 0.01).*

### Metabolic Differences between Young and Old Leaves of Cotton Treated with Alkali Stress

**FIGURE 4:**
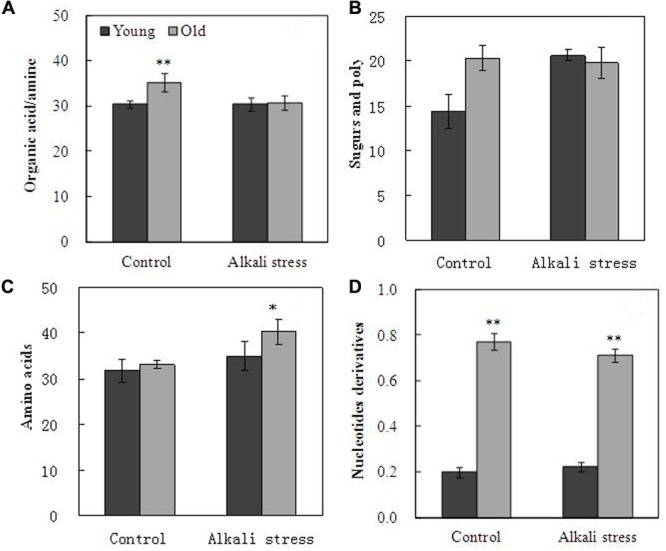
**Effects of alkali stress on the total relative concentration of organic acids/amine (A)**, sugars and poly-sugars **(B)**, amino acids **(C)**, and nucleotide derivatives **(D)** in young and old leaves of cotton seedlings. The values are the means ± SE of five biological replicates, and each replicate consisted of a pool of five plants. Significant difference between organs under similar stress condition was determined by *T*-test and marked as ^∗^*P* < 0.05 and ^∗∗^*P* < 0.01. The seedlings were subjected to 80 mM alkali stress (NaHCO_3_:Na_2_CO_3_ = 9:1; pH = 9.06) for 6 days.

Alkali stress strongly affected the metabolites, including organic acids, amino acids, sugars and poly-sugars, and nucleotide derivatives in leaves (**Figure 4**). Alkali stress had little effect on young leaves but it considerably increased sugar and poly-sugar contents (**Figure 4**). In addition, alkali stress significantly reduced the amounts of organic acids but it increased amino acid contents in old leaves (**Figure 4**). In the TCA cycle, the amounts of oxalic acid, citric acid, aconitic acid, α-ketoglutaric acid, and succinic acid significantly decreased, and the degree of reduction was higher in old leaves than in young leaves under alkali stress treatment (**Table 2**). Mannose was up-accumulated in cotton in addition to the metabolites involved in glycolysis and which were obviously up-accumulated in young leaves (**Table 2**). Glutamic acid (used in proline synthesis) was depleted dramatically probably because of the synthesis of downstream metabolites; proline increased by 5.02- and 2.62-folds in young and old leaves under alkali stress (**Table 2**). In glutamine synthetase/synthase (GS/GOGAT), the amounts of transamination-related metabolites clearly changed in young leaves under alkali stress; asparagine contents increased, whereas aspartic acid and glutamine contents decreased (**Table 2**). GABA pathway was enhanced under alkali stress, resulting in increased GABA and putrescine contents (**Table 2**). Moreover, the concentration decline of ethanolamine and increase of glycine and serine is indicative that the metabolism of plasma membrane was inhibited (**Table 2**). Shikimic acid pathway in young leaves was not significantly affected by alkali stress. However, the amount of shikimic acid significantly decreased and that of quinic acid significantly increased in old leaves, demonstrating that shikimic acid pathway was inhibited in old leaves under alkali stress (**Table 2**).

## Discussion

### Growth

The young and old leaves of cotton exposed to 80 mM alkali stress for 6 days showed distinct differences in terms of growth; alkali stress exerted a stronger inhibitory effect on old leaves than on young leaves. This result implies that young organs are protected at the expense of the old organs; this phenomenon is possibly an adaptive strategy of plants to alkali stress. Alkali stress considerably increased Na^+^ content of both old and young leaves; however, old leaves accumulated higher Na^+^ concentration than young leaves (**Table 1**). Moreover, alkali stress did not significantly influence K^+^ content of young leaves but strongly reduced K^+^ content in old leaves (**Table 1**). Na^+^ enters the plant cells via the K^+^ transporter pathways and through the non-selective cation channels, and Na^+^ exclusion mechanism is dependent on H^+^ gradient across cell membrane (Zhu, 2003; Yang et al., 2012). These results maybe indicated that cotton could compartmentalize Na^+^ into vacuoles of old leaves to prevent the onset of ion toxicity in the entire young organ. Cotton plants possibly utilize a specific regulatory mechanism of Na^+^ transmission in old leaves. The results indicated that alkali stress exerted no significant effect on Ca, Mg, Fe, Cu, Zn, and Mn contents in both leaves, and this result was possibly caused by the short-term treatment with 80 mM alkali stress (**Table 1**). Accumulation of large amounts of Na^+^ in tissue causes ionic imbalance; to maintain ionic balance and pH homeostasis, plants usually accumulate inorganic anions, such as Cl^-^, NO_3_^-^, and SO_4_^2-^ (Yang et al., 2008, 2009). Our results showed that old and young leaves accumulate Cl^-^, indicating that Cl^-^ accumulation is a general adaptive response to excessive Na^+^ in cotton leaves (**Figure 2**). However, under alkali stress, SO_4_^2-^ content was increased in young leaves but descreased in old leaves. This phenomenon is possibly a special adaptive response to excessive Na^+^ in young leaves and possibly plays important roles in maintenance of ionic balance and pH homeostasis in young leaves (**Figure 2**).

### Metabolites

Plants growing in saline environments suffer from osmotic stress and are exposed to excessive Na^+^, which induces generation of reactive oxygen species (ROS) and causes protease activation and intracellular hyperammonemia (Parida and Das, 2005; Zhang J.T. et al., 2011). To avoid accumulation of excessive Na^+^ and hyperammonemia-induced cytotoxicity, plant cells normally react through ion transport, compartmentation, synthesis of compatible solutes, and transamination metabolites, which is abundant in plant tissues and involved in active synthesis and metabolism of energy (Zhang J.T. et al., 2011; Yang et al., 2012). However, interference between salinity and metabolites is a very complex network affecting nearly all metabolic and developmental processes in plants.

Metabolic changes caused by alkali stress (80 mM) are dependent on age of tissues (**Supplementary File S1**). Proline plays as osmoprotectant in plants subjected to drought or salinity stress (Delauney and Verma, 1993; Hare and Cress, 1997). Under alkali stress, young leaves and old leaves increased 2^5^- and 2^2^-fold (fold was calculated by the formula: log_2_^Alkali/control^) in proline content compared to their controls (**Table 2**). The same phenomenon in cultivated cotton leaves was reported by Hu et al. (2009) and Chen et al. (2011). Moreover, reduction in glutamic acid contents indicated that glutamic acid was converted into proline by Δ1-pyrroline-5-carboxylate synthase (P5CS), especially in young leaves. Sugars, the amount of which increased significantly in young leaves under alkali stress, are compatible solutes produced in response to salinity stress (Chen and Murata, 2002). The amounts of inositol and mannose in both old and young leaves significantly increased in cotton subjected under alkali stress, and the extent of increase was higher in young leaves than in old leaves. Inositol plays important functions in membrane biosynthesis, and they protect the membrane by acting as free radical scavengers; our result is similar to that for *Actinidia* (kiwifruit) leaves subjected under salinity conditions as reported by Klages et al. (1999). Organic acid synthesis was significantly inhibited in old leaves. Citric acid, succinic acid, and malic acid, which are effective ROS-scavenging metabolites, were significantly downregulated in old leaves. By contrast, synthesis of amino acids, such as GABA, valine, and serine, was dramatically enhanced in old leaves, and this phenomenon is favorable for osmotic adjustment and membrane stability. The results suggest that proline is a common compatible solute that showed great changes under alkali stress in leaves; in addition, sugars (glucose, inositol, and mannose) were the compatible solutes specific in young leaves, whereas some amino acids (GABA, valine, and serine) were the solutes specific in old leaves. Under alkali stress, *TCA* cycle was inhibited significantly in old leaves compared with that in young leaves. The results suggest that alkali stress (high pH) caused mass dissipation of energy, and organic acid synthesis was inhibited in old leaves, implying that alkali stress strongly influenced the energy production in old leaves. Under alkali stress, young leaves showed high amounts of photosynthetic pigments and accumulation of increased number of metabolites (PEP, 3PGA, F6P, G6P, and glucose) that are associated with glycolysis. This finding suggests that alkali stress stimulated production of reducing force and enhanced N metabolism, which in turn increased sugar production and enhanced glycolysis in young leaves. In *GS/GOGAT* cycle, aspartic acid and glutamine contents decreased and asparagine content increased in young leaves under alkali stress, suggesting that these changes in the amounts of transamination-related metabolites are consistent with the shift of metabolic activities toward proline biosynthesis. *GABA* pathway apparently converts transamination products into compatible osmolytes (Shelp et al., 2006). In the present case, *GABA* contents increased dramatically in cotton leaves, indicating that *GABA* functioned as osmoregulator and as cytosolic pH regulator in response to alkali stress; moreover, *GABA* serves as an intermediate that assists in biosynthesis of osmolytes, such as myo-inositol and proline (Bouche and Fromm, 2004). Ethanolamine and shikimic acid are plant metabolites that participate in synthesis of membrane phospholipids, phosphatidylethanolamines, and lignin, which are used in the synthesis of plant cell membranes and cell wall (McNeil et al., 1999; Wang et al., 2002). Our results suggested that suppression of ethanolamine and shikimic acid restricts cell membrane and wall elongation, leading to growth inhibition of old leaves of cotton under alkali stress.

In summary, alkali stress exerted a considerably stronger limiting effect on old leaves than on young leaves. Under alkali stress, young leaves can maintain low Na and high K contents, as well as maintain a relatively stable pigment accumulation and TCA, resulting in increased accumulation of photosynthetic metabolites. Comparison of the metabolic response of the old and young leaves clearly shows that they display different mechanisms of osmotic regulation. Under stress condition, organs at different developmental stages possibly utilize different mechanisms of metabolic regulation. We suggest that future investigations on alkali stress should include more organs obtained at different development stages.

## Author Contributions

RG and LS designed the study. RG, CWY, QL, and XX performed the experiments. RG, XZ, CRY and HL analyzed the data. RG wrote the manuscript. All authors read and approved the final manuscript.

## Conflict of Interest Statement

The authors declare that the research was conducted in the absence of any commercial or financial relationships that could be construed as a potential conflict of interest.

## Footnotes

1 http://www.genome.jp/kegg/

2 www.metaboanalyst.ca/

3 http://www.plantcyc.org/

## Supplementary Material

FILE S1 **Relative concentration and fold changes of 133 metabolites in young and old leaves of cotton seedlings after 6 days of alkali stress treatment.** The relative concentration of each metabolite is an average of data from five biological replicates using GC-MS. The fold changes was calculated using the formula log_2_^(treatment/control)^. ^∗^ Indicate significant (*P* < 0.05) and ^∗∗^ indicate highly significant (*P* < 0.01), respectively.

FILE S2 **Relative concentration and fold changes of 133 metabolites in young and old leaves of cotton seedlings after 6 days of alkali stress treatment**.
