# Supplementary material for: Comparison of Ionomic and Metabolites Response under Alkali Stress in Old and Young Leaves of Cotton (Gossypium hirsutum L.) Seedlings
Source: Front Plant Sci. 2016 Nov 25;7:1785. doi: 10.3389/fpls.2016.01785 (PMC5122583; doi:10.3389/fpls.2016.01785)
Supplement: FILE S1 — Relative concentration and fold changes of 133 metabolites in young and old leaves of cotton seedlings after 6 days of alkali stress treatment. The relative concentration of each metabolite is an average of data from five biological replicates using GC-MS. The fold changes was calculated using the formula log2(treatment/control). ∗ Indicate significant (P < 0.05) and ∗∗ indicate highly significant (P < 0.01), respectively. [file Table_1.DOC]

File 1. Relative concentration and fold changes of 133 metabolites in young and old leaves of cotton seedlings after 6 days of 80mM alkali stress treatment. The relative concentration of each metabolite is an average of data from five biological replicates using GC-MS. The fold changes was calculated using the formula log_2_^(treatment/control)^. * and ** indicate significant (*P*<0.05) and highly significant difference (*P*<0.01), respectively.

| Metabolite | CK | | AS | | log_2_^AS/CK^ | |
| --- | --- | --- | --- | --- | --- | --- |
|  | Young | Old | Young | Old | Young  Leaves | Old  Leaves |
|  | Mean CK-Y | Mean CK-O | Mean AS-Y | Mean AS-O |  |  |
| Oxalic acid | 0.06 | 0.03 | 0.05 | 0.01 | -0.32 | -0.96* |
| Citric acid | 1.21 | 2.09 | 1.16 | 1.02 | -0.06 | -1.04* |
| Aconitic acid | 0.21 | 0.03 | 0.10 | 0.01 | -1.02* | -1.34** |
| α-ketoglutaric acid | 0.28 | 0.19 | 0.16 | 0.10 | -0.82 | -0.91* |
| Succinic acid | 4.67 | 2.95 | 2.98 | 0.94 | -0.65 | -1.65** |
| Fumaric acid | 0.19 | 0.55 | 0.19 | 0.43 | 0.02 | -0.35 |
| Malic acid | 5.15 | 12.18 | 6.42 | 8.27 | 0.32 | -0.56 |
| Pyruvic acid | 0.17 | 0.29 | 0.19 | 0.31 | 0.14 | 0.09 |
| PEP | 0.01 | 0.03 | 0.03 | 0.02 | 1.12* | -0.24 |
| 3PGA | 0.16 | 0.22 | 1.03 | 0.65 | 2.66** | 1.55* |
| Fructose-6-phosphate | 0.02 | 0.03 | 0.05 | 0.03 | 0.98* | 0.24 |
| Glucose-6-phosphate | 0.03 | 0.05 | 0.07 | 0.04 | 0.95* | -0.28 |
| Glucose | 0.02 | 0.22 | 0.11 | 0.20 | 2.18** | -0.12 |
| Fructose | 1.86 | 2.40 | 2.42 | 2.03 | 0.38 | -0.24 |
| Sucrose | 0.18 | 0.51 | 0.14 | 0.30 | -0.36 | -0.78* |
| Galactose | 0.08 | 0.02 | 0.09 | 0.04 | 0.25 | 1.09* |
| γ-aminobutyric acid | 2.50 | 6.04 | 3.96 | 8.72 | 0.67* | 0.53* |
| Succinate semialdehyde | 0.01 | 0.01 | 0.01 | 0.01 | -0.22 | -0.52 |
| Putrescine | 0.29 | 0.03 | 0.45 | 0.04 | 0.64* | 0.28 |
| Alanine | 2.80 | 5.69 | 2.19 | 3.55 | -0.35 | -0.68* |
| Aspartic acid | 14.35 | 16.16 | 9.94 | 18.41 | -0.53* | 0.19 |
| Asparagine | 0.10 | 0.12 | 0.67 | 0.12 | 2.68** | -0.02 |
| Glutamine | 0.19 | 0.22 | 0.10 | 0.15 | -1.00* | -0.58 |
| Glycine | 0.18 | 0.15 | 0.16 | 0.29 | -0.20 | 0.89 |
| Serine | 0.67 | 0.69 | 0.45 | 0.92 | 0.04 | 1.05* |
| Ethanolamine | 4.52 | 2.03 | 4.33 | 0.82 | -0.06 | -1.32* |
| Shikimic acid | 0.95 | 0.30 | 0.72 | 0.15 | -0.41 | -1.02* |
| Quinic acid | 0.57 | 0.01 | 1.11 | 0.02 | 0.97* | 1.18* |
| Phenylalanine | 0.70 | 1.11 | 1.27 | 1.45 | 0.85 | 0.39 |
| Cinnamic acid | 0.46 | 0.14 | 0.65 | 0.14 | 0.50 | 0.04 |
| Ferulic acid | 0.00 | 0.01 | 0.00 | 0.01 | 0.30 | -0.03 |
| Chlorogenic acid | 0.43 | 0.01 | 0.72 | 0.02 | 0.75 | 0.62 |
| Tyrosine | 0.16 | 0.06 | 0.24 | 0.07 | 0.57 | 0.14 |
| Tryptophan | 0.39 | 0.28 | 0.45 | 0.48 | 0.20 | 0.79 |
| myo-inositol | 3.35 | 3.59 | 7.55 | 5.74 | 1.17* | 0.68 |
| Xylose | 0.03 | 0.10 | 0.04 | 0.11 | 0.16 | 0.05 |
| Xylitol | 0.47 | 0.05 | 0.40 | 0.03 | -0.24 | -1.12* |
| Trehalose | 0.01 | 0.01 | 0.01 | 0.01 | 0.47 | 0.26 |
| Threitol | 0.04 | 0.15 | 0.04 | 0.14 | -0.30 | -0.09 |
| Tagatose | 0.12 | 0.13 | 0.28 | 0.41 | 1.19* | 1.63** |
| Sorbitol | 0.73 | 0.67 | 0.21 | 0.59 | -1.81* | -0.18 |
| Sedoheptulose | 0.02 | 0.01 | 0.01 | 0.01 | -0.34 | 0.06 |
| Ribose-5-phosphate | 0.01 | 0.00 | 0.01 | 0.00 | 0.34 | -3.09 |
| Ribose | 0.27 | 0.36 | 0.53 | 0.29 | 1.00* | -0.30 |
| Ribitol | 0.04 | 0.08 | 0.02 | 0.10 | -0.78 | 0.27 |
| Raffinose | 0.03 | 0.09 | 0.01 | 0.07 | -0.84 | -0.48 |
| Piceatannol | 0.08 | 0.01 | 0.08 | 0.00 | 0.14 | -1.16* |
| Phytosphingosine | 0.01 | 0.01 | 0.00 | 0.01 | -1.30* | 0.91 |
| Phytol | 0.07 | 0.20 | 0.13 | 0.78 | 0.87 | 1.97** |
| Mannose | 2.51 | 2.02 | 4.77 | 3.48 | 0.92 | 0.78 |
| Mannitol | 0.05 | 0.19 | 0.04 | 0.16 | -0.41 | -0.25 |
| Maltotriose | 0.03 | 0.10 | 0.05 | 0.12 | 0.63 | 0.18 |
| Maltotriitol | 0.00 | 0.00 | 0.00 | 0.00 | 0.69 | -0.28 |
| Maltose | 0.01 | 0.22 | 0.03 | 0.22 | 1.16* | -0.02 |
| Lyxose | 0.00 | 0.02 | 0.01 | 0.01 | 0.67 | -1.78* |
| Threose | 0.03 | 0.03 | 0.03 | 0.02 | -0.04 | -0.49 |
| Levoglucosan | 0.00 | 0.01 | 0.00 | 0.01 | -1.04* | 0.14 |
| Lactulose | 0.02 | 0.06 | 0.02 | 0.05 | 0.25 | -0.29 |
| Lactose | 0.00 | 0.01 | 0.00 | 0.00 | 0.36 | -1.51* |
| Glucose-1-phosphate | 0.34 | 0.28 | 0.32 | 0.27 | -0.10 | -0.02 |
| Gentiobiose | 0.01 | 0.05 | 0.01 | 0.05 | 0.42 | -0.13 |
| Galactinol | 0.69 | 0.50 | 0.52 | 0.47 | -0.41 | -0.07 |
| Fucose | 0.09 | 0.32 | 0.08 | 0.21 | -0.10 | -0.61 |
| Farnesol | 1.42 | 0.50 | 1.28 | 0.40 | -0.16 | -0.31 |
| Erythrose | 0.13 | 0.28 | 0.22 | 0.14 | 0.77 | -1.00* |
| Dodecanol | 0.01 | 0.01 | 0.01 | 0.02 | 0.06 | 1.31* |
| Dithioerythritol | 0.01 | 0.03 | 0.03 | 0.02 | 1.75* | -0.19 |
| D-erythro-sphingosine | 0.04 | 0.09 | 0.03 | 0.08 | -0.43 | -0.17 |
| Arabitol | 0.09 | 0.05 | 0.04 | 0.08 | -1.00* | 0.77 |
| Altrose | 0.00 | 0.00 | 0.00 | 0.02 | -1.20* | 1.73* |
| Cellobiose | 0.04 | 0.09 | 0.02 | 0.12 | -0.78 | 0.39 |
| Allose | 0.19 | 2.02 | 0.18 | 1.61 | -0.10 | -0.33 |
| Kestose | 0.29 | 0.10 | 0.63 | 0.07 | 1.12* | -0.58 |
| Glycerol | 0.76 | 0.87 | 1.00 | 0.38 | 0.39 | -1.18* |
| Diglycerol | 0.10 | 0.33 | 0.09 | 0.34 | -0.21 | 0.07 |
| Valine | 0.40 | 0.75 | 0.86 | 1.89 | 1.09* | 1.33* |
| Threonine | 0.15 | 0.17 | 0.16 | 0.45 | 0.14 | 1.41* |
| Proline | 1.04 | 2.73 | 33.99 | 16.79 | 5.02** | 2.62** |
| Methionine | 0.08 | 0.03 | 0.02 | 0.12 | -1.69* | 2.05** |
| Cysteine | 0.01 | 0.01 | 0.01 | 0.02 | 0.34 | 0.24 |
| Isoleucine | 0.19 | 0.40 | 0.87 | 1.26 | 2.20** | 1.65* |
| Glutamic acid | 0.06 | 0.08 | 0.03 | 0.09 | -1.02* | -0.17 |
| Cycloleucine | 0.10 | 0.07 | 0.09 | 0.03 | -0.20 | -1.36* |
| Citrulline | 0.02 | 0.06 | 0.01 | 0.04 | -0.91 | -0.82 |
| Alanine | 0.01 | 0.00 | 0.02 | 0.01 | 0.51 | 1.46* |
| Uridine | 0.16 | 0.69 | 0.20 | 0.64 | 0.35 | -0.12 |
| Uracil | 0.01 | 0.01 | 0.01 | 0.02 | -0.30 | 1.60* |
| Thymine | 0.00 | 0.00 | 0.00 | 0.00 | 0.11 | 2.29** |
| Thymidine | 0.01 | 0.04 | 0.01 | 0.01 | -0.32 | -1.52* |
| Guanosine | 0.01 | 0.00 | 0.00 | 0.00 | -2.68** | -0.90 |
| Cytosin | 0.00 | 0.00 | 0.00 | 0.00 | -1.32* | 1.45* |
| Adenosine | 0.01 | 0.04 | 0.00 | 0.03 | -1.88* | -0.17 |
| Threonic acid | 1.27 | 0.30 | 1.05 | 0.37 | -0.27 | 0.30 |
| Terephthalic acid | 0.01 | 0.01 | 0.01 | 0.01 | -0.74 | 0.49 |
| Tartaric acid | 0.03 | 0.04 | 0.04 | 0.04 | 0.31 | -0.08 |
| Saccharic acid | 1.41 | 0.23 | 2.12 | 0.17 | 0.58 | -0.43 |
| Pipecolinic acid | 0.50 | 0.94 | 1.36 | 1.08 | 1.45* | 0.20 |
| Phenylpyruvate | 0.10 | 0.10 | 0.07 | 0.05 | -0.58 | -0.88 |
| Pelargonic acid | 0.01 | 0.01 | 0.01 | 0.01 | -0.36 | -0.37 |
| Palmitic acid | 0.38 | 0.61 | 0.39 | 0.56 | 0.03 | -0.11 |
| Oleic acid | 0.02 | 0.01 | 0.02 | 0.01 | -0.43 | 0.61 |
| Nicotinic acid | 0.01 | 0.10 | 0.05 | 0.10 | 1.82* | 0.01 |
| Mucic acid | 0.30 | 0.10 | 0.23 | 0.09 | -0.41 | -0.24 |
| Methylmalonic acid | 0.01 | 0.01 | 0.01 | 0.01 | 0.11 | -0.77 |
| Malonic acid | 0.38 | 0.13 | 0.33 | 0.17 | -0.20 | 0.38 |
| Maleic acid | 0.01 | 0.08 | 0.01 | 0.03 | -1.37* | -1.44* |
| Linolenic acid | 0.06 | 0.09 | 0.04 | 0.06 | -0.63 | -0.53 |
| Linoleic acid | 0.07 | 0.01 | 0.02 | 0.02 | -1.47* | 1.42* |
| Lactobionic acid | 0.00 | 0.02 | 0.00 | 0.02 | 1.18* | -0.28 |
| Lactic acid | 0.79 | 1.68 | 0.90 | 2.34 | 0.18 | 0.48 |
| Itaconic acid | 0.00 | 0.00 | 0.00 | 0.00 | 0.49 | 0.14 |
| Heptadecanoic acid | 0.00 | 0.01 | 0.00 | 0.01 | 0.35 | 0.21 |
| Glycolic acid | 0.06 | 0.03 | 0.09 | 0.05 | 0.57 | 0.51 |
| Glucuronic acid | 0.08 | 0.03 | 0.09 | 0.03 | 0.06 | 0.11 |
| Glucoheptonic acid | 0.31 | 0.10 | 0.41 | 0.17 | 0.37 | 0.78 |
| Gallic acid | 0.10 | 0.98 | 0.14 | 0.75 | 0.41 | -0.38 |
| Galactonic acid | 1.87 | 3.25 | 2.01 | 3.64 | 0.10 | 0.17 |
| Glyceric acid | 0.23 | 0.38 | 0.36 | 0.80 | 0.63 | 1.08* |
| Galacturonic acid | 0.02 | 0.00 | 0.03 | 0.01 | 1.06 | 1.38* |
| Dehydroascorbic acid | 1.42 | 1.71 | 0.91 | 0.89 | -0.64 | -0.94* |
| Citramalic acid | 0.10 | 0.06 | 0.14 | 0.06 | 0.43 | -0.10 |
| Citraconic acid | 0.00 | 0.01 | 0.00 | 0.01 | -0.30 | -1.14 |
| Gondoic acid | 0.00 | 0.00 | 0.00 | 0.00 | 0.19 | -0.24 |
| Caffeic acid | 0.25 | 0.15 | 0.38 | 0.15 | 0.59 | 0.04 |
| Glycerophosphoric acid | 0.08 | 0.05 | 0.07 | 0.03 | -0.33 | -0.79 |
| Azelaic acid | 0.00 | 0.00 | 0.00 | 0.00 | 0.35 | 1.74* |
| Ascorbate | 0.09 | 0.07 | 0.09 | 0.05 | 0.11 | -0.39 |
| Aminooxyacetic acid | 1.30 | 2.64 | 1.17 | 1.52 | -0.15 | -0.80* |
| Aminomalonic acid | 0.01 | 0.00 | 0.01 | 0.00 | -0.28 | -0.90 |
| 6-phosphogluconic acid | 0.00 | 0.00 | 0.00 | 0.00 | -0.10 | -0.90 |
| Butanoic acid | 0.03 | 0.18 | 0.06 | 0.10 | 0.89 | -0.90 |
| Furoic acid | 0.06 | 0.05 | 0.11 | 0.07 | 0.81 | 0.37 |
| Glycerol-1-phosphate | 0.06 | 0.04 | 0.08 | 0.03 | 0.27 | -0.36 |
