# Supplementary figures and images for: Comparison of Ionomic and Metabolites Response under Alkali Stress in Old and Young Leaves of Cotton (Gossypium hirsutum L.) Seedlings
Source: Front Plant Sci. 2016 Nov 25;7:1785. doi: 10.3389/fpls.2016.01785 (PMC5122583; doi:10.3389/fpls.2016.01785)

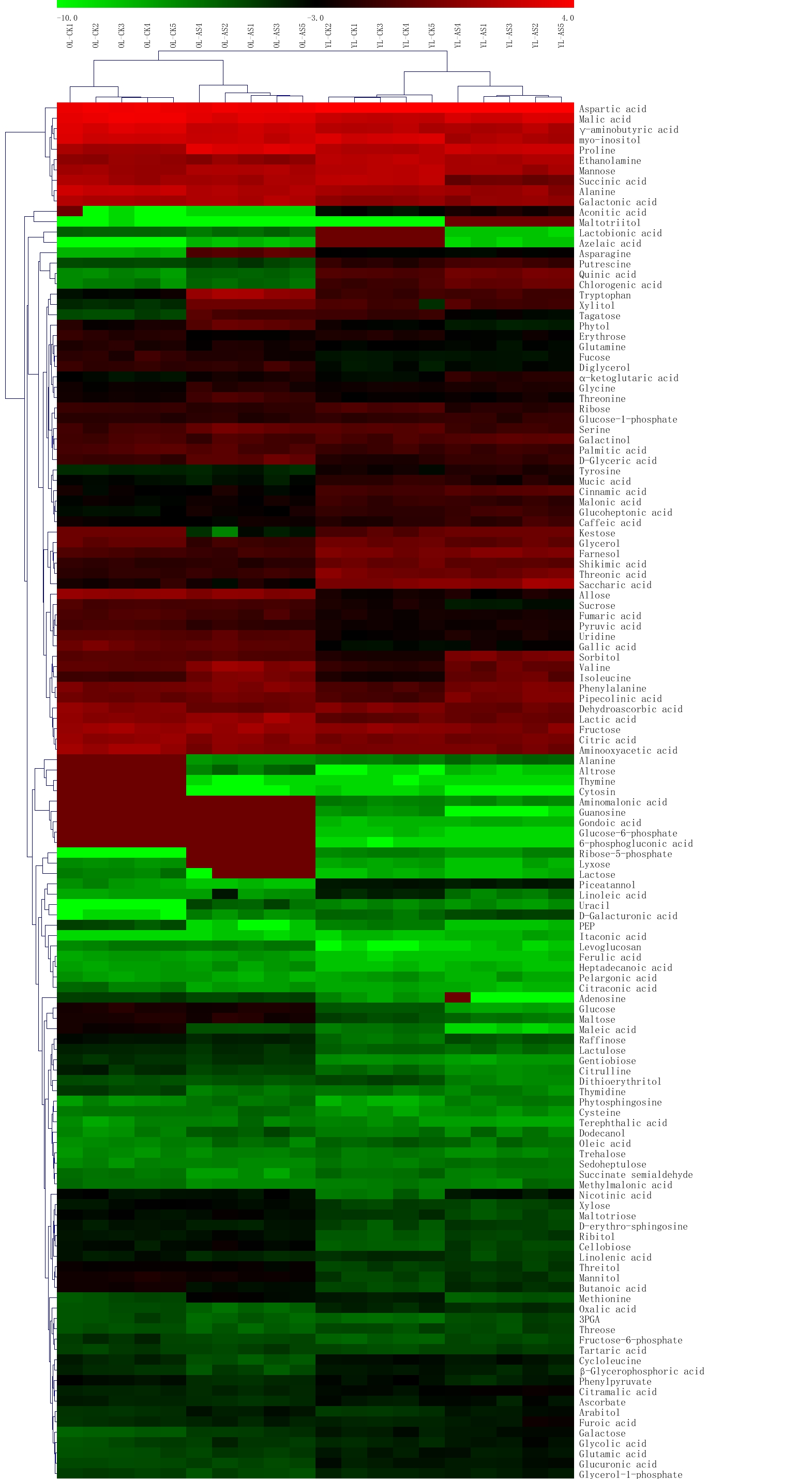

Supplement: FILE S2 — Relative concentration and fold changes of 133 metabolites in young and old leaves of cotton seedlings after 6 days of alkali stress treatment. [file Image_1.JPEG]
